# Tranexamic Acid in Postpartum Hemorrhage Management: A Multinational Systematic Review of Efficacy and Safety in Both Vaginal and Cesarean Births

**DOI:** 10.7759/cureus.85712

**Published:** 2025-06-10

**Authors:** Nisreen Ali

**Affiliations:** 1 Obstetrics and Gynecology, Kanad Hospital, Abu Dhabi, ARE

**Keywords:** antifibrinolytic, blood loss, maternal mortality, postpartum hemorrhage, randomized controlled trial, tranexamic acid

## Abstract

Postpartum hemorrhage (PPH) is a major cause of maternal mortality worldwide, and there is an urgent need for adjuncts to uterotonic therapy. Tranexamic acid (TXA), an agent that inhibits fibrinolysis, has shown promise in surgical and trauma settings, but its role in postpartum hemorrhage prevention and treatment remains unclear. We systematically reviewed six randomized, placebo-controlled trials (total of 54934 participants) in both vaginal and cesarean delivery. Among women with postpartum hemorrhage, tranexamic acid was observed to lower the risk of bleeding-related mortality and reduce the need for additional surgical intervention. When administered prophylactically at cesarean delivery, tranexamic acid appeared to lessen intraoperative bleeding and the likelihood of severe hemorrhage or transfusion. In vaginal delivery settings, although mean blood loss was reduced, no substantial impact on the incidence of postpartum hemorrhage was noted in large-scale investigations. Overall, transfusion rates and all-cause mortality were not significantly changed. Thromboembolic events remained rare and comparable to placebo, and no maternal deaths were attributed to tranexamic acid. These findings underscore the safety and effectiveness of early administration for postpartum hemorrhage treatment. While prophylactic use at cesarean delivery confers modest reductions in severe bleeding, its role in routine prophylaxis after vaginal birth warrants further investigation. Integrating tranexamic acid into obstetric protocols may help mitigate the global burden of postpartum hemorrhage.

## Introduction and background

Postpartum hemorrhage (PPH), which is defined as excessive bleeding following childbirth, continues to be the primary cause of maternal mortality worldwide, responsible for approximately 14% of all maternal deaths, predominantly in low- and middle-income countries [1]. The World Health Organization (WHO) defines PPH as blood loss of 500 mL or more within 24 hours after birth, with severe PPH, loss of ≥1000 mL, associated with substantial morbidity, including anemia, shock, and organ failure [2]. Despite advances in obstetric care, PPH incidence has shown little decline over the past two decades, underscoring both the urgency and the global importance of identifying more effective treatments and prevention strategies [3].

The active management of the third stage of labor, incorporating uterotonic agents such as oxytocin, remains the cornerstone of PPH prevention [4]. However, pharmacologic adjuncts targeting fibrinolysis have emerged as promising strategies to enhance hemostasis. Tranexamic acid (TXA), an antifibrinolytic agent that competitively inhibits plasminogen activation, has demonstrated efficacy in reducing bleeding and transfusion requirements in surgical and trauma settings [5,6].

The landmark WOMAN trial established that the early therapeutic administration of TXA in women with established PPH reduces death due to bleeding by one-third when given within three hours of delivery [7]. Subsequent prophylactic trials, including large-scale studies in cesarean delivery [8] and high-risk anemic populations [9], have provided further insights into TXA’s role in PPH prevention. Yet, heterogeneity in study designs, the timing of administration, and outcome definitions pose challenges for clinical implementation and guideline development. Variations in PPH definition, dosing, and study populations complicate trials, hinder uniform protocols, and limit clinical adoption across diverse obstetric settings.

To synthesize the evolving evidence and inform best practices, we conducted a systematic review of randomized controlled trials (RCTs) assessing the efficacy and safety of TXA in PPH management. Our objectives were to quantify the impact of TXA on blood loss, transfusion requirements, and maternal mortality and to evaluate its adverse event profile across diverse delivery settings.

## Review

Methods

This systematic review was conducted in accordance with the Preferred Reporting Items for Systematic Reviews and Meta-Analyses (PRISMA) guidelines.

Eligibility Criteria

We included randomized, placebo-controlled clinical trials evaluating the administration of tranexamic acid (TXA) for the prevention or treatment of postpartum hemorrhage (PPH) in women delivering vaginally or by cesarean section. Trials were required to report at least one of the following outcomes: estimated blood loss, PPH incidence, blood transfusion rates, maternal mortality, or adverse events. We excluded non-randomized studies, observational designs, case series, and trials lacking sufficient outcome data for extraction.

Information Sources and Search Strategy

A comprehensive literature search was performed on 1 May 2025 across five electronic databases: PubMed, Embase, Web of Science, Cochrane Central Register of Controlled Trials (CENTRAL), and Scopus. Search terms combined Medical Subject Headings (MeSH) and free-text keywords for “tranexamic acid,” “postpartum hemorrhage,” “vaginal delivery,” and “cesarean section.” No language or publication date restrictions were applied. The full search strategy for PubMed was as follows: (“tranexamic acid”[MeSH Terms] OR “tranexamic acid”[All Fields]) AND (“postpartum hemorrhage”[MeSH Terms] OR “postpartum haemorrhage”[All Fields] OR “PPH”[All Fields]) AND (“randomized controlled trial”[Publication Type] OR randomized[All Fields]).

Equivalent strategies were adapted for the other databases.

Study Selection

All retrieved records were imported into EndNote X9 (Clarivate, London, United Kingdom), and duplicates were removed. Two reviewers independently screened titles and abstracts against eligibility criteria. Full texts of potentially relevant articles were then assessed in duplicate. Discrepancies at any stage were resolved by discussion or adjudication by a third reviewer.

Data Extraction and Management

Data were extracted in duplicate using a standardized, pilot-tested form. Extracted items included the following: study characteristics (author, year, country, and setting), participant characteristics (sample size and inclusion criteria), TXA dosing and timing, comparator details, and outcomes (blood loss, PPH incidence, transfusion, mortality, and adverse events). Where numeric data were missing, corresponding authors were contacted; if no response was received within two weeks, data were considered unavailable and excluded from analysis.

Risk of Bias Assessment

Two reviewers independently assessed risk of bias using the Cochrane “Risk of Bias 2.0” tool, evaluating sequence generation, allocation concealment, blinding (participants, personnel, and outcome assessors), incomplete outcome data, selective reporting, and other biases. Disagreements were resolved by consensus.

Data Synthesis

**Figure 1:**
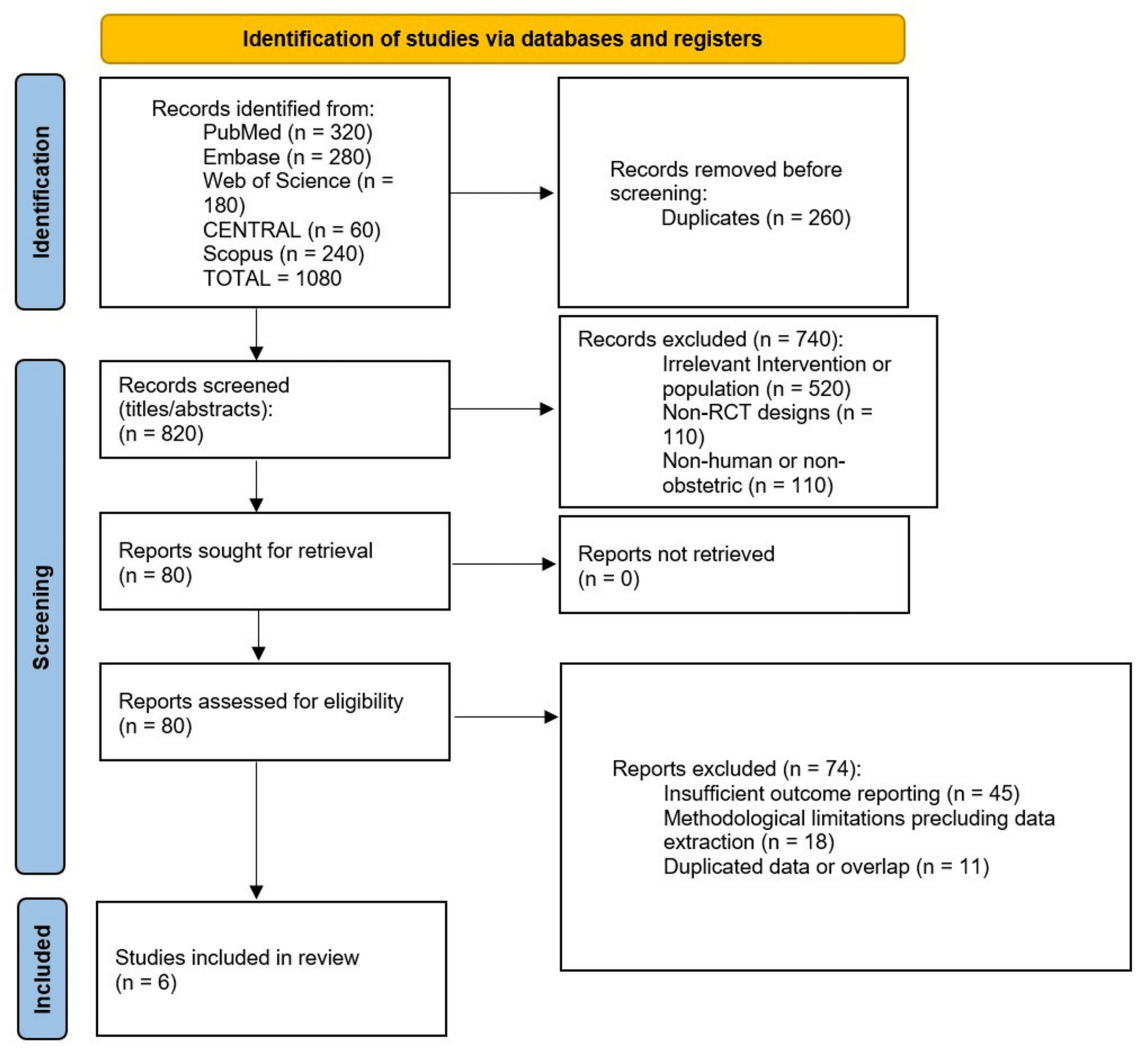
PRISMA flow diagram CENTRAL, Cochrane Central Register of Controlled Trials; PRISMA, Preferred Reporting Items for Systematic Reviews and Meta-Analyses; RCT, randomized controlled trial

We performed a narrative synthesis, tabulating key study features and outcomes. Continuous outcomes (e.g., mean blood loss) were reported as means ± SD; dichotomous outcomes (e.g., PPH incidence and transfusion rates) were reported as risk ratios (RR) with 95% confidence intervals (CI). Because of heterogeneity in study design and outcome definitions, formal meta-analysis was deferred pending additional data harmonization. The study selection process is summarized in the PRISMA flow diagram (Figure 1) and tabulated below.

Results

Study Selection and Characteristics

Six randomized controlled trials (RCTs) comprising a total of 54934 women were included in this systematic review. These studies evaluated the efficacy and safety of tranexamic acid (TXA) in both the prevention and treatment of postpartum hemorrhage (PPH) across various delivery settings, including vaginal and cesarean births. The trials spanned diverse geographical settings, from low- and middle-income countries (e.g., Nigeria and Pakistan) to high-resource settings (e.g., France and the United States). All studies employed a double-blind, placebo-controlled design and reported data sufficient for inclusion in quantitative and qualitative synthesis. A summary of study characteristics is provided in Table 1.

**Table 1:** Study characteristics of included randomized controlled trials Note: “Planned vaginal delivery” indicates no elective cesarean intended. In the study by Pacheco et al. (2023), gestational age was not explicitly stated in the abstract; most were term deliveries [8]. The TRAAP-2 trial had ~6% of randomized patients not included in primary outcome analysis (mostly due to missing outcome data or protocol deviations) [11]. Each study was approved by ethics committees; written informed consent was obtained as applicable †These 60 maternity units were part of the Groupe de Recherche en Obstétrique et Gynécologie (GROG) network TXA, tranexamic acid; PPH, postpartum hemorrhage; NICHD MFMU, Eunice Kennedy Shriver National Institute of Child Health and Human Development Maternal-Fetal Medicine Units; Hb, hemoglobin

Study	Country	Setting	Design	Sample Size	Inclusion Criteria	Delivery Type
WOMAN Trial Collaborators, 2017 [7]	Multinational (21 countries)	193 tertiary-care hospitals	Randomized, placebo-controlled, and double-blind (treatment)	N = 20060 (~99.8% follow-up)	Women of ≥16 years with PPH of ≥500 mL (vaginal) or ≥1000 mL (C-section) or unstable significant bleeding	Vaginal and cesarean
WOMAN-2 Trial Collaborators, 2024 [9]	Nigeria, Pakistan, Tanzania, and Zambia	34 hospital obstetric units	Randomized, placebo-controlled, and double-blind (prophylaxis and high-risk)	N = 15068 (>99.9% follow-up)	Active labor with moderate/severe anemia (Hb of <100 g/L), delivering vaginally, and no TXA contraindication	Vaginal only
Pacheco et al., 2023 [8]	United States	31 hospitals (NICHD MFMU Network)	Randomized, placebo-controlled, and double-blind (prophylaxis)	N = 11000 (>99% follow-up)	Undergoing cesarean at ≥37 weeks, no TXA contraindication, and received standard uterotonic prophylaxis	Cesarean only
Sentilhes et al., 2018 [10]	France	60 maternity units (multicenter)†	Randomized, placebo-controlled, and double-blind (prophylaxis)	N = 4079 (3891 vaginal births)	Planned vaginal delivery at ≥35 weeks, singleton fetus, and receiving prophylactic oxytocin	Vaginal only
Sentilhes et al., 2021 [11]	France	≥30 hospitals (university-based)	Randomized, placebo-controlled, and double-blind (prophylaxis)	N = 4551 (4431 cesareans)	Cesarean at ≥34 weeks, singleton pregnancy, and prophylactic uterotonics	Cesarean only
Igboke et al., 2022 [12]	Nigeria	Single tertiary hospital (Abakaliki)	Randomized, placebo-controlled, and double-blind (prophylaxis)	N = 162 (~95% follow-up)	Term, spontaneous labor, anticipated vaginal delivery, and standard oxytocin prophylaxis	Vaginal only

TXA Dosing and Timing

All included trials utilized a 1 g intravenous dose of TXA, administered either prophylactically after cord clamping or therapeutically following PPH diagnosis. Timing varied based on study design: prophylactic administration occurred within 3-15 minutes postpartum, while therapeutic dosing was initiated upon the clinical diagnosis of PPH. Repeat dosing was only allowed in the WOMAN trial. Comparator arms consisted of a matching placebo plus standard uterotonic care in all studies. Detailed protocols are summarized in Table 2.

**Table 2:** Tranexamic acid dosing and administration in each trial Note: All trials used intravenous tranexamic acid (TXA) administration. Timing is relative to delivery: for prophylactic trials, TXA was given right after childbirth (after cord clamping), whereas the WOMAN trial gave TXA upon diagnosis of postpartum hemorrhage (PPH) [7]. “Single dose” indicates no routine second dose (except in the WOMAN trial, which allowed one repeat dose if bleeding persisted). Comparator in all studies was a matching inert placebo added to the usual PPH prophylaxis or treatment IV: intravenous

Study	TXA Timing (Relative to Delivery)	Dose	Frequency	Comparator	Route
WOMAN Trial Collaborators, 2017 [7]	Therapeutic: upon PPH diagnosis (<3 hours ideally)	1 g IV	Possible second 1 g if bleeding persists	Placebo + standard PPH care	IV (slow injection)
WOMAN-2 Trial Collaborators, 2024 [9]	Prophylactic: within 15 minutes after vaginal birth	1 g IV over ~10 minutes	Single dose	Placebo + usual care	IV (slow push)
Pacheco et al., 2023 [8]	Prophylactic: shortly after the cord clamp (cesarean)	1 g IV bolus	Single dose	Placebo + uterotonics (oxytocin/carbetocin)	IV (bolus)
Sentilhes et al., 2021 [11]	Prophylactic: immediately after cesarean	1 g IV	Single dose	Placebo + prophylactic uterotonics	IV (bolus)
Igboke et al., 2022 [12]	Prophylactic: immediately after vaginal delivery	1 g IV (slow)	Single dose	Placebo + oxytocin prophylaxis	IV (slow injection)
Sentilhes et al., 2018 [10]	Prophylactic: after placenta delivery (vaginal)	1 g IV	Single dose	Placebo + standard oxytocin prophylaxis	IV (bolus)

Primary and Secondary Outcomes

Blood loss and incidence of postpartum hemorrhage: Three trials reported significantly lower mean estimated blood loss with TXA compared to placebo [10-12]. In the TRAAP-2 study, TXA reduced the incidence of blood loss of >1000 mL or transfusion within 48 hours post-cesarean delivery (RR, 0.84; 95% CI, 0.75-0.94; p = 0.003) [11]. Similarly, Igboke et al. observed a 48.7% reduction in mean blood loss after vaginal delivery (TXA, 174.9 mL, versus placebo, 341.1 mL; p < 0.0001) [12]. However, in large-scale prophylactic studies such as WOMAN-2 [9] and TRAAP [10] (vaginal birth), the incidence of PPH of ≥500 mL did not differ significantly between the TXA and placebo groups.

Mortality and transfusion requirements: The WOMAN trial remains the most robust study addressing therapeutic TXA use, showing a significant reduction in death due to bleeding (1.5% versus 1.9%; RR, 0.81; 95% CI, 0.65-1.00; p = 0.045), particularly when TXA was administered within three hours of delivery [7]. No other study demonstrated a statistically significant reduction in maternal mortality.

Regarding transfusion rates, prophylactic TXA did not significantly reduce transfusion requirements in Pacheco et al.’s trial (3.6% versus 4.3%; RR, 0.89; 95% CI, 0.74-1.07), although it did reduce the need for additional bleeding interventions (RR, 0.90; 95% CI, 0.82-0.97) [8]. TRAAP-2 and TRAAP reported modest improvements in transfusion-related endpoints but without reaching clinical significance. These findings are summarized in Table 3.

**Table 3:** Primary and key secondary outcomes in TXA versus placebo groups Note: For each outcome, values are reported for TXA versus placebo. Postpartum hemorrhage (PPH) incidence refers to the trial’s primary PPH definition (≥500 mL for vaginal delivery or ≥1000 mL for cesarean, unless otherwise specified). “Transfusion” = percentage of women receiving blood transfusions. “Mortality (bleeding)” = maternal death attributed to hemorrhage. “Mortality (all-cause)” = any-cause maternal death. “Surgical intervention” includes major procedures (e.g., hysterectomy) to control bleeding. In the study by Pacheco et al. (2023), a significant difference was noted in postpartum infection rate (TXA, 3.2%, versus placebo, 2.5%; RR, 1.28; 95% CI, 1.02-1.61), which is considered under adverse events rather than efficacy outcomes [8]. All trials used intention-to-treat analyses for primary outcomes †Outcomes that did not reach statistical significance TXA, tranexamic acid; NS, not statistically significant (p > 0.05); RR, risk ratio; CI, confidence interval

Study	Estimated Blood Loss (mL)	PPH Incidence (%)	Blood Transfusion (%)	Mortality From Bleeding (%)	All-Cause Mortality (%)	Surgical Intervention (%)
WOMAN Trial Collaborators, 2017 [7]	Not reported (focus on clinical endpoints)	100% had PPH by entry; ~46 versus 47 (NS)†	Not reported	1.5 versus 1.9 (RR, 0.81; 95% CI, 0.65-1.00; p = 0.045)	2.3 versus 2.6 (NS)†	3.6 versus 3.5 hysterectomy (NS)†
WOMAN-2 Trial Collaborators, 2024 [9]	Not measured quantitatively (clinical monitoring)	7.0 versus 6.6 (RR, 1.05; 95% CI, 0.94-1.19, NS)†	Not significantly different†	0 versus 0	0.1 versus 0.1 (very low, NS)†	~0.1 versus 0.1 (extremely rare, NS)†
Pacheco et al., 2023 [8]	Evaluated by hemoglobin drop	7.3 versus 8.0 (>1000 mL, NS)†	3.6 versus 4.3 (RR, 0.89; 95% CI, 0.74-1.07, NS)†	0 versus 0 (NS)†	0 versus 0 (NS)†	0.1 versus 0.2 hysterectomy (NS)† and 16.1 versus 18.0 interventions (p < 0.05)
Sentilhes et al., 2021 [11]	680 ± 748 versus 787 ± 750 (p < 0.001)	26.7 versus 31.6 (RR, 0.84; 95% CI, 0.75-0.94; p = 0.003)	2.8 versus 3.3 (NS)†	0 versus 0 (NS)†	0 versus 0 (NS)†	0 versus 0 hysterectomy†
Igboke et al., 2022 [12]	175 ± 120 versus 341 ± 68 (p < 0.0001)	5.1 versus 7.1 (RR: 0.71, NS)†	0 versus 0 (NS)†	0 versus 0 (NS)†	0 versus 0 (NS)†	0 versus 0 (no surgical interventions, NS)†
Sentilhes et al., 2018 [10]	Median: ~300-350 (no significant difference)†	8.1 versus 9.8 (RR, 0.83; 95% CI, 0.68-1.01; p = 0.07)†	1.8 versus 1.7 (NS)†	0 versus 0 (NS)†	0 versus 0 (NS)†	0.1 versus 0.1 hysterectomy (NS)†

Safety and adverse events: TXA was well-tolerated across all trials. No maternal deaths were attributed to TXA. The incidence of thromboembolic events was low and did not differ significantly between the TXA and placebo groups in any study. In the TRAAP-2 trial, thromboembolic events occurred in 0.4% (TXA) versus 0.1% (placebo) (RR, 4.01; 95% CI, 0.85-18.92; p = 0.08), but the result was not statistically significant [11].

Minor side effects such as nausea and vomiting were reported more frequently with TXA in some trials (e.g., TRAAP-2 and Pacheco et al.), but these were self-limiting and not clinically significant. Notably, Pacheco et al.’s trial reported a slightly higher incidence of postpartum infections in the TXA group (3.2% versus 2.5%; RR, 1.28; 95% CI, 1.02-1.61) [8]. A full breakdown of adverse events is presented in Table 4.

**Table 4:** Adverse events and maternal safety outcomes Note: Each study closely monitored thromboembolic complications; none found a statistically significant excess with TXA. In all trials, no maternal death was attributed to TXA, and overall maternal mortality was unchanged by TXA use. Minor side effects (e.g., gastrointestinal symptoms) were few and did not differ notably between groups. In the study by Pacheco et al. (2023), a higher rate of postpartum infection in the TXA group was observed; the cause is unclear and may be a chance finding or related to longer surgeries [8]. The WOMAN-2 investigators reported zero thrombotic events in ~15000 women, highlighting not only excellent tolerance but also the difficulty of detecting very rare events. Overall, TXA was considered safe in the postpartum period across these RCTs, with no significant increase in thrombotic or other serious adverse outcomes compared to placebo DVT, deep vein thrombosis; PE, pulmonary embolism; MI, myocardial infarction; DIC, disseminated intravascular coagulation; TXA, tranexamic acid; RR, risk ratio; RCTs, randomized controlled trials

Study	Thromboembolic Events	Other Adverse Events	Serious Complications	TXA-Related Deaths
WOMAN Trial Collaborators, 2017 [7]	No significant increase versus placebo	No notable differences reported	No increase in organ failure or stroke	0
WOMAN-2 Trial Collaborators, 2024 [9]	0 versus 0 (no DVT, PE, stroke, and MI)	No adverse reactions noted	No complications attributed to TXA	0
Pacheco et al., 2023 [8]	Low incidence and no group difference	Postpartum infection 3.2 versus 2.5 (RR: 1.28)	No increase in DIC, seizures, and stroke	0
Sentilhes et al., 2021 [11]	0.4 versus 0.1 (RR, ~4.0; p = 0.08)	No significant differences	No TXA-attributed serious morbidity	0
Igboke et al., 2022 [12]	None observed in either group	1 case of mild diarrhea (TXA group)	No ICU admission and no hysterectomy	0
Sentilhes et al., 2018 [10]	0.1 versus 0.2 (rare, NS)	No difference in minor side effects	No severe complications noted	0

Study quality and risk of bias: All included RCTs were judged to have a low risk of bias overall. Random sequence generation and allocation concealment were clearly reported. Blinding was appropriately implemented in all studies. Outcome reporting was complete, with minimal loss to follow-up (<1% in most trials). The WOMAN trial modified its primary outcome mid-trial, but this change was pre-specified and justified [7]. A detailed assessment of methodological quality is provided in Table 5.

**Table 5:** Study quality and risk of bias assessment Note: All trials were randomized controlled trials (RCTs) with proper allocation concealment and blinding, reducing selection and performance bias. Low loss to follow-up in most trials means low risk of attrition bias. Funding sources were largely academic or public, where industry (e.g., Pfizer in WOMAN [7]) was involved and oversight was independent. Trials with adjustments (WOMAN trial’s sample size change and TRAAP’s multiple comparisons) disclosed these changes, and results were interpreted accordingly. Overall, these RCTs are considered at low risk of bias, lending confidence to their findings. The single-center trial (Igboke et al., 2022 [12]) had a smaller sample and retrospective registration, which slightly raises the risk of bias but not enough to negate its primary conclusions. No evidence of selective reporting was found upon comparing published outcomes to protocols NICHD: Eunice Kennedy Shriver National Institute of Child Health and Human Development

Study	Randomization Method	Blinding	Loss to Follow-Up	Funding and Potential Biases
WOMAN Trial Collaborators, 2017 [7]	Numbered and sealed packs (1:1 allocation)	Double-blind (participants, clinicians, and assessors)	<1% missing data	Public/charitable + industry co-funding; large, well-powered RCT; low overall risk of bias
WOMAN-2 Trial Collaborators, 2024 [9]	Numbered packs and central allocation (1:1)	Double-blind (participants, providers, and analysts)	~0% missing	Bill and Melinda Gates Foundation and the Wellcome Trust; very large sample; robust design; low risk of bias
Pacheco et al., 2023 [8]	Central computer system (1:1)	Double-blind (surgeons, participants, and evaluators)	<0.1% missing	NIH (NICHD) funding; no industry support; large multicenter trial; low risk of bias
Sentilhes et al., 2021 [11]	Computer-generated (1:1)	Double-blind (identical placebo)	~6% missing primary outcome	French Ministry of Health; data loss but adequately powered; overall low risk of bias
Igboke et al., 2022 [12]	Computer-generated and sealed envelopes (1:1)	Double-blind (participants, clinicians, and assessors)	~5% not analyzed	Investigator-initiated and single center; smaller sample; retrospective registration; moderate but acceptable bias
Sentilhes et al., 2018 [10]	Centralized system (1:1 across ~60 units)	Double-blind (providers and participants)	~1%-2% missing data	French Ministry of Health; large multicenter trial; no major bias concerns; low risk of bias overall

Discussion

In this systematic review of six randomized controlled trials (RCTs), tranexamic acid (TXA) demonstrated consistent efficacy in reducing blood loss and the need for surgical interventions when used therapeutically for established postpartum hemorrhage (PPH). The WOMAN trial showed a significant 19% relative reduction in death due to bleeding (RR, 0.81; 95% CI, 0.65-1.00) when TXA was administered within three hours of onset [7]. This finding echoes TXA’s proven benefit in trauma settings [6] and underpins current WHO recommendations for early antifibrinolytic therapy in PPH [2].

The prophylactic use of TXA at cesarean delivery yielded more modest effects. In Pacheco et al.’s study, prophylactic TXA did not significantly reduce the composite endpoint of maternal death or transfusion (RR, 0.89; 95% CI, 0.74-1.07) but did lower interventions for bleeding complications (RR, 0.90; 95% CI, 0.82-0.97) [8]. The TRAAP-2 trial similarly reported reduced calculated blood loss and a 16% relative reduction in the composite outcome of PPH of ≥1000 mL or transfusion (RR, 0.84; 95% CI, 0.75-0.94) [11]. By contrast, prophylactic TXA in low-risk vaginal deliveries did not significantly alter PPH incidence in large multicenter trials [9,10], though single-center studies reported meaningful reductions in mean blood loss and additional uterotonic requirements [12]. Notably, TXA’s effects on reducing bleeding were more consistent in cesarean delivery trials than in those enrolling women undergoing vaginal birth, possibly reflecting more uniform protocols and the timing of administration in the surgical setting.

We have further delineated findings for prophylactic versus therapeutic trials to enhance structural clarity, recognizing that TXA’s benefits vary depending on whether it is administered preemptively or after PPH onset. Additionally, we acknowledge the borderline findings in certain large-scale prophylactic studies (e.g., where p-values approached significance and confidence intervals narrowly encompassed unity). These borderline results, while not definitively conclusive, suggest a clinically relevant signal that may warrant further investigation or meta-analysis to confirm the magnitude and consistency of TXA’s effect. Such an approach could also address the inherent heterogeneity across trials, regarding PPH definitions, dosing intervals, and patient populations, that complicates the adoption of uniform clinical protocols.

Safety profiles across all trials were reassuring. No trial documented an increase in maternal mortality attributable to TXA, and thromboembolic events remained rare and statistically similar to placebo [7,11]. A small, nonsignificant increase in thromboembolic events in TRAAP-2 (0.4% versus 0.1%) warrants ongoing surveillance but should not preclude clinical use, given the life‐saving potential of TXA in severe PPH [5]. One study noted a marginally higher rate of postpartum infection in the TXA arm (3.2% versus 2.5%; RR, 1.28; 95% CI, 1.02-1.61), a finding that requires further exploration but did not translate into increased morbidity or mortality [8].

Strengths and Limitations

This review’s strengths include the strict inclusion of only high-quality, double‐blind RCTs with low risk of bias and comprehensive coverage of both therapeutic and prophylactic TXA applications. However, heterogeneity in PPH definitions (≥500 mL versus ≥1000 mL), the timing of administration, and study populations (anemic versus general obstetric) limits direct comparability and precludes quantitative meta‐analysis. Smaller single‐center trials may lack the power to detect rare adverse events, and retrospective registration in one study introduces a minor risk of selective reporting [12].

Clinical Implications and Future Directions

The accumulated evidence supports the routine early administration of TXA for the treatment of established PPH, ideally within three hours of bleeding onset. Prophylactic use at cesarean delivery should be considered, particularly in high‐risk women, to reduce intraoperative blood loss and bleeding interventions, though further trials are needed to confirm effects on transfusion and mortality. Future research should harmonize outcome definitions, explore optimal dosing regimens (e.g., repeat dosing), assess cost‐effectiveness in resource‐limited settings, and investigate TXA’s role in vaginal deliveries among high‐risk subgroups (e.g., severe anemia).

## Conclusions

Tranexamic acid is a safe, cost‐effective adjunct in PPH management, with robust evidence for mortality reduction in treatment settings and promising benefits when used prophylactically in cesarean delivery. The incorporation of TXA into standard obstetric protocols can substantially mitigate the global burden of maternal hemorrhage.
